# Systematic Analysis of NB-ARC Gene Family in Rice and Functional Characterization of *GNP12*

**DOI:** 10.3389/fgene.2022.887217

**Published:** 2022-06-16

**Authors:** Ying-Hua Pan, Lei Chen, Hai-Feng Guo, Rui Feng, Qi-Jin Lou, Muhammad Abdul Rehman Rashid, Xiao-Yang Zhu, Dong-Jin Qing, Hai-Fu Liang, Li-Jun Gao, Cheng-Cui Huang, Yan Zhao, Guo-Fu Deng

**Affiliations:** ^1^ Rice Research Institute, Guangxi Academy of Agricultural Sciences/Guangxi Key Laboratory of Rice Genetics and Breeding, Nanning, China; ^2^ State Key Laboratory of Agrobiotechnology/Beijing Key Laboratory of Crop Genetic Improvement, College of Agronomy and Biotechnology, China Agricultural University, Beijing, China; ^3^ Department of Bioinformatics and Biotechnology, Government College University Faisalabad, Faisalabad, Pakistan; ^4^ Guangxi Academy of Agricultural Sciences/Guangxi Crop Genetic Improvement and Biotechnology Laboratory, Nanning, China; ^5^ State Key Laboratory of Crop Biology, Shandong Key Laboratory of Crop Biology, College of Agronomy, Shandong Agricultural University, Tai’an, China

**Keywords:** NB-ARC family, evolution, expression, panicle development, rice

## Abstract

The NB-ARC (nucleotide-binding adaptor shared by APAF-1, R proteins, and CED-4) gene family plays a critical role in plant development. However, our understanding of the mechanisms of how NB-ARC genes regulate plant development in the plant panicle is still limited. Here, we subjected 258 NB-ARC genes in rice to genome-wide analysis to characterize their structure, function, and expression patterns. The NB-ARC genes were classified into three major groups, and group II included nine subgroups. Evolutionary analysis of NB-ARC genes in a dicotyledon plant (*Arabidopsis thaliana*) and two monocotyledonous plants (*Oryza sativa* L. and *Triticum aestivum*) indicated that homologous genome segments were conserved in monocotyledons and subjected to weak positive selective pressure during evolution. Dispersed and proximal replication events were detected. Expression analysis showed expression of most NB-ARC genes in roots, panicles, and leaves, and regulation at the panicle development stage in rice Ce253. The *GNP12* gene encodes RGH1A protein, which regulates rice yield according to panicle length, grain number of panicle, and grain length, with eight major haplotypes. Most members of NB-ARC protein family are predicted to contain P-loop conserved domains and localize on the membrane. The results of this study will provide insight into the characteristics and evolution of NB-ARC family and suggest that *GNP12* positively regulates panicle development.

## Introduction

NB-ARC (nucleotide-binding adaptor shared by APAF-1, R proteins, and CED-4) proteins are critical regulators of signaling pathways and play important roles in effector recognition and signal transduction in healthy plant growth and development (Jacquemin et al., 2014; Wen et al., 2017; Chen et al., 2018). NB-ARC proteins contain an NB-ARC domain that is proposed to act as a molecular switch, with a nucleotide-binding (NB) subdomain including a P-loop, a C-terminal extension that forms a four-helix bundle (ARC1), and a winged-helix fold (van der Biezen and Jones, 1998; Albrecht and Takken, 2006). The NB subdomain can bind and hydrolyze ATP *in vitro* (Tameling et al., 2002). NB-ARC belongs to the STAND (signal transduction ATPases with numerous domains) family of NTPases, and is proposed to work as an NTP-hydrolyzing switch by binding and hydrolyzing NTP and regulating signal transduction by conformational changes (Leipe et al., 2004). Recent studies revealed NB-ARC proteins with similar central nucleotide-binding-ARC domain architectures act in metazoan innate immunity and programmed cell death pathways (Slootweg et al., 2009). For example, NB-ARC genes share homology with human APAF-1 and *C. elegans* CED-4, proteins that regulate cell death (Yildirim-Ersoy et al., 2011). *RPP1A* belongs to the NB-ARC gene family and reduces plant growth with broad-spectrum resistance to virulent strains of *H. parasitica* in *Arabidopsis* (Michael Weaver et al., 2006). Constitutive expression of TIR-NB-ARC-LRR gene *VpTNL1* in *Arabidopsis* resulted in either a wild-type or dwarf phenotype (Wen et al., 2017). NB-ARC genes might contribute to *R. glutinosa* consecutive monoculture problems (Chen et al., 2018). Domains can work together as a platform to mediate downstream signal transduction events (Lukasik and Takken, 2009). In wheat, many NB-ARC gene clusters include highly similar genes likely formed by tandem duplications (Andersen et al., 2020). Some NB genes have been reported to be regulated in response to morphological development (Igari et al., 2008). *AhRAF4* was observed to correlate with morphological changes in development in maize (Dolezal et al., 2014). NB-ARC-containing sequences in wheat exhibit maximum homology with proteins from *indica* and *Brachypodium distachyon*, and distributions of NB-ARC sequences are balanced among the three wheat sub-genomes (Chandra et al., 2017). In *indica*, *Oryza glaberrima* (Slootweg et al., 2009), and *Oryza brachyantha* (FF), conserved NB-ARC genes were subjected to strong purifying selection but selection was more relaxed for expanded homologous genes (Jacquemin et al., 2014). NB-ARC protein function has been studied in wheat, rice, and *Arabidopsis*, but the mechanism of NB-ARC protein function in rice panicle development remains poorly understood.

Rice is one of the most important cereal crops in the world and feeds half population of the world (Zhao et al., 2015). In recent years, many important genes controlling rice grain yield have been isolated and functionally characterized. *LP1* encodes a Remorin_C-containing protein of unknown function and the *LP1* allele of Xiushui79 leads to reduced panicle length (Liu et al., 2016). *Grain Number per Panicle1* (*GNP1*), Rice *GA20ox1* encoding a cytokinin (Tameling et al., 2002), and the biosynthesis gene for gibberellins upregulate cytokinin activity to increase grain number and grain yield in rice (Wu et al., 2016). *GW8* encodes an SBP-domain transcription factor that regulates grain width by binding directly to the *GW7* promoter to repress its expression (Wang et al., 2015). *OsMKK3* encodes a MAP kinase that controls rice grain size and chalkiness (Pan et al., 2021). Although few studies have focused on NB-ARC proteins in rice, these important plant proteins likely have multiple regulatory roles. For example, the gain-of-function mutation of NB-ARC protein RLS1 (Rapid Leaf Senescence1) causes high-light-dependent HR-like cell death in rice (Wang et al., 2020). Overall, a greater understanding of mechanisms underlying grain yield will strengthen our understanding of regulatory mechanisms for these traits and facilitate the breeding of crop varieties with high-yield potential.

In this study, members of the NB-ARC gene family were identified from analysis of the rice genome. The motif composition and gene structures of these genes were systematically analyzed, and tandem duplication and gene replication events were identified. Collinear relationships between rice, wheat*,* and *Arabidopsis* were compared. The expression levels of 11 subgroups of NB-ARC genes in different tissues and panicle development of rice Ce253 were analyzed by RNA-Seq and the expression levels of 18 genes were measured via qPCR. Functional analysis was performed of a selected NB-ARC gene, *GNP12*. This gene serves as a positive regulator in panicle development and panicle length. The results of this research provide expand our understanding of the function of the NB-ARC gene family and provide guidance for future efforts to improve rice breeding.

## Materials and Methods

### Rice Materials

The rice variety Ce253, widely planted in Guangxi Province, China, was selected for this study and was obtained from the Rice Research Institute, Guangxi Academy of Agricultural Sciences. Rice was planted under natural field conditions at the Rice Research Institute of Guangxi Academy of Agricultural Sciences, Nanning, China in 2020. The distance between plants within rows was 16 cm, and the distance between plants in separate rows was 20 cm. Field management, including irrigation, fertilizer application, and pest control, were performed according to normal agricultural practices. Fully filled grains were subjected to grain width, length, and weight measurement with a Wanshen SC-G automatic seed test system. All trait measurements were repeated at least three times.

### Identification of Gene Family Members

Genome-wide identification of NB-ARC genes from three species of monocotyledonous and dicotyledonous plants was performed. Hidden Markov Model (HMM) (Punta et al., 2012) (version 3.0) analysis was used for the search. HMM profiles of NB-ARC genes (PF00931.2) were obtained from the Pfam database (http://pfam.xfam.org/) with an e-value≤1e-3. The results of the HMMER sequence alignment were screened to remove protein sequences that were at least 45% longer than the length of the HMM model domain, while retaining the longest protein sequence in the variable shear. Simple Modular Architecture Research Tool (SMART) (version 8.0) (http://smart.embl-heidelberg.de) was used for further analysis with all non-redundant protein sequences (Schultz et al., 1998). Finally, 258 NB-ARC genes models were identified in the rice genome for further analysis. The basic information of the identified NB-ARC proteins was obtained using the tools at the ExPasy website (http://web.expasy.org/protparam/). CDS coordinate information is listed in Supplementary Table S1. Gene family data were analyzed by Gene Denovo Biotechnology Co., Ltd. (Guangzhou, China). A BLASTP search of the NCBI nonredundant protein database was used to assign the NB-ARC domains. Multiple alignments of NB-ARC domains with 11 different plant NB-ARC domains of 11 subgroups were performed using MEME. The submotifs were analyzed through http://weblogo.berkeley.edu/logo.cgi. The 3D model of the NB-ARC genes was predicted by Jpred and SWISS-MODEL. We used the intersection results of Pfam (pfoo31, - e 1e-20) + smart (- e 0.1 -- dome 0.01) and blast (- e 1e-14 and identity >28%) as the identified homologous genes.

### Gene Structure Analysis, Chromosomal Distribution, and Gene Duplication

The exon-intron structural information for the collected rice NB-ARC genes was acquired from reference genome annotation files (Os-Nipponbare-Reference-IRGSP-1.0 pseudomolecules) to compare genomic and coding sequences (http://rice.uga.edu/). The cDNAs were aligned with their corresponding genomic DNA sequences. To map all NB-ARC genes, the chromosome distribution and conserved regions were confirmed by analysis of reference genome annotation files. A chromosome distribution diagram was drawn using the SVG package in Perl. Gene duplication in rice was identified using the replicate gene classifier program of MCScanX software (Wang et al., 2012). All protein-coding sequences were aligned using blastp, and the alignment results were used as input files for MCScanX software to predict gene replication. A gene was identified as a replication gene according to e-value<1e-5 or e-value<1e-10. Five replication events were identified: segmental, tandem, proximal, and decentralized (dispersed).

### Motifs, Phylogenetic, Combination Diagram, and Closely Related Species Analysis

Conserved motifs in the gene family sequences were identified using the MEME program (http://alternate.meme-suite.org/tools/meme) with statistical significance (Bailey et al., 2015). The MEME program was run with default settings, maximum motif search value of 15, and an optimum motif width of 10–100 amino acid residues. A phylogenetic tree was constructed with the neighbor-joining algorithm in MEGA (version 7.0) with bootstrap test of 1,000 times and drawn with iTOL (https://itol.embl.de/) (Kumar et al., 2016). ML (maximum likelihood method) evolutionary tree was showed in Supplementary Figure S1. Genome annotations and corresponding protein sequences were downloaded from EnsemblPlants (*Brassica*_rapa. IVFCAAS v1.36, *Brassica*_oleracea.v2.1.36, *Arabidopsis*_*thaliana*.TAIR10) and GenoScope (*Brassica*_napus.annotation_v5). The gene structure and motifs were analyzed by systematic evolutionary relationships. Synteny detection was performed by McScanX and drawn with Circos software.

### Ka/Ks Analysis

We compared the Ka/Ks ratios as a proxy of the selective pressures acting on gene pairs for reciprocal best match gene pairs from *Arabidopsis*, rice, and wheat*.* Ka/Ks was calculated as the ratio of the number of nonsynonymous substitutions per non-synonymous site (Ka) in a period to the number of synonymous substitutions per synonymous site (Ks) in the same period. Ka, Ks, and Ka/Ks values are based on coding sequence alignment and calculated using the KaKs_calculator software package based on the Nei and Gojobori model (Nei and Gojobori, 1986). Positively or negatively selected sites were identified based on Ka/Ks ratios with a confidence interval for each ratio given by a *p* value and an adjusted *p* value (Adj.Pval) from multiple comparisons (Massingham and Goldman, 2005). Homologous genes with a Ka/Ks ratio above 1 were under strong positive selection, between 0.5 and 1 were considered to be under weak positive selection, and below 0.1 were considered to be under negative selection (purifying selection).

### Vector Construction and Rice Transformation and Subcellular Localization

To assess subcellular localization, sgRNA-Cas9 plant expression vectors were constructed as described previously (Mao et al., 2013). The targeting sequence (5′- TAG​TCG​ACG​ACA​ATG​CTG​CCA​GG-3′), corresponded to +400 to +422 within the third exon encoding the C-terminal end of *GNP12* (starting at amino acid residue 134). To construct the GFP plasmids, the cDNAs of *LOC_Os101g458510* were amplified from Nipponbare and the cDNA for *LOC_Os12g36720* was amplified from Ce253. The cDNAs were cloned into the pBWA(V)HS-ccdb-GLos-GFP vector to generate the insertion. Plasmids CaMV35S:GFP, CaMV35S:*LOC_Os101g458510*-GFP and CaMV35S:*GNP12*-GFP were transformed into rice leaf protoplasts for subcellular localization. GFP was excited with a 488 nm laser and imaging was performed.

### DNA Extraction, RNA Extraction, Expression Analysis, and RNA-Seq

Panicles of Ce253 of different lengths (3, 5, 10, 15, 20, and 25 cm) at the booting stage were flash-frozen in liquid nitrogen. Total RNA was extracted from these tissues using a EasyPure^®^ Plant RNA Kit (Trans, Catalog no. ER301-01). RNA purity was determined by assaying 1 µl of total RNA extract on a NanoDrop 1,000. We measured the optical density (OD) ratio between 260 and 280 nm from samples, where pure RNA eluted in H20 (pH 7.0–8.5) or TE (pH 8.0) is expected to exhibit a ratio of 2.0–2.1. Total RNA (2.0 μg) was used for cDNA synthesis with a PrimeScript^™^ RT Reagent Kit with gDNA Eraser (TransScript^®^ II One-Step RT-PCR SuperMix, Tran, Catalog no. AH411-02). The resulting cDNA samples were diluted five-fold and used as templates for qRT-PCR using a TransStart^®^ Green qPCR SuperMix and a CFX96 RealTime system (Bio-RAD, Hercules, CA, United States) following the manufacturer’s instructions. The qRT-PCR reactions were performed as 10 µl mixtures containing 5 µl of 2× Green qPCR MasterMix, 1 µl of cDNA, 0.25 µl of each primer (10 µM), and 3.5 µl of ddH_2_O. Amplification steps were 95°C for 30 s, 40 cycles of 95°C for 5 s, and 60°C for 30 s, followed by 65°C for 5 s, 95°C for 15 s, 60°C for 30 s, and 95°C for 15 s. Each experiment was repeated at least three times. The qRT-PCR analysis was performed using the ΔΔCt method. Details on gene-specific primers used for real-time PCR are provided in Supplementary Table S1. The ubiquitin gene (*LOC_Os03g13170*) (Chen et al., 2019) was used as a control (^*^ *p* < 0.05; ^**^ *p* < 0.01; Student’s *t-*test). RNA samples used for RNA-seq analysis were prepared from different panicles of Ce253 grown under normal field conditions with three biological replicates. RNA library sequencing was performed on an Illumina Hiseq™ 2,500/4,000 platform by Majorbio. Sequence analysis was performed using the method provided by Majorbio (http://www.majorbio.com/). RNA-Seq data for NB-ARC genes in different tissues were obtained from http://expression.ic4r.org/.

### Haplotype and Evolutionary Analysis

The single-nucleotide polymorphisms (SNPs) of 827 accessions used for haplotype analysis were acquired from the 3K rice genomes (3K-RG) dataset. The grain length data for 827 accessions were downloaded from the Rice SNP-Seek Database. One-way ANOVA followed by Duncan’s new multiple-range test were performed using SPSS 21.0 software. The genomic sequences of 1,400 cultivated and 58 wild accessions were obtained from the 3K rice genomes (3K-RG) dataset and OryzaGenome (http://viewer.shigen.info/oryzagenome/), respectively, and were used to construct a minimum spanning tree for RGH1A. Arlequin version 3.5 software was used to calculate a haplotype network and the distance matrix output was used in Hapstar-0.6 to draw a minimum spanning tree. The average nucleotide diversity (π) and Tajima’s D for each subpopulation in RGH1A and 40-kb flanking regions were calculated using DnaSP 5.10 software (Librado and Rozas, 2009). The nucleotide diversity curves were generated using 60 bp window and 15 bp step length.

## Results

### NB-ARC Genes in Rice Were Analyzed and Subgroup IIf Was Identified as a Representative Subgroup

**FIGURE 1:**
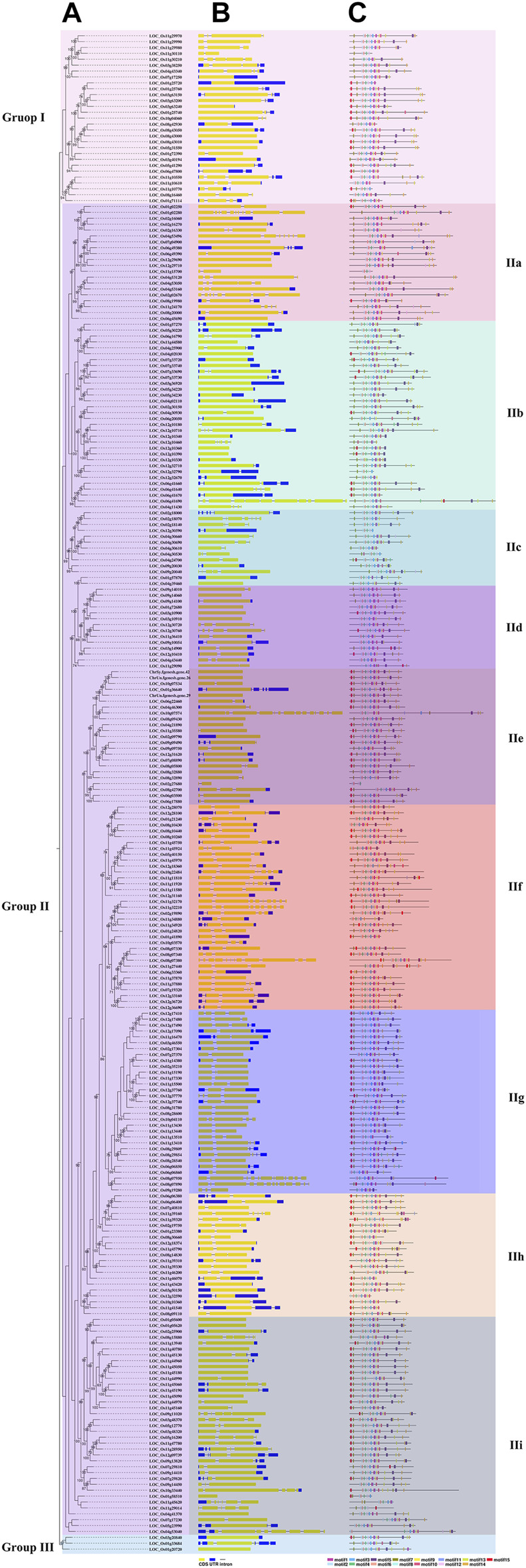
Phylogenetic tree representing the relationships among 258 genes of rice. Phylogenetic tree **(A)**, exon/intron structure. Number is bootstrap values. **(B)**, and motif composition **(C)** of NB-ARC genes in rice. Neighbor-Joining (NJ)-Phylogenetic trees shown in a and b were prepared using the same methods. The widths of Gy bars at the bottom indicate relative lengths of genes and proteins. Yellow boxes and blue lines in b represent exons and introns, respectively. Different boxes in c represent different motifs. Different background colors represent different groups and subgroups.

**FIGURE 2:**
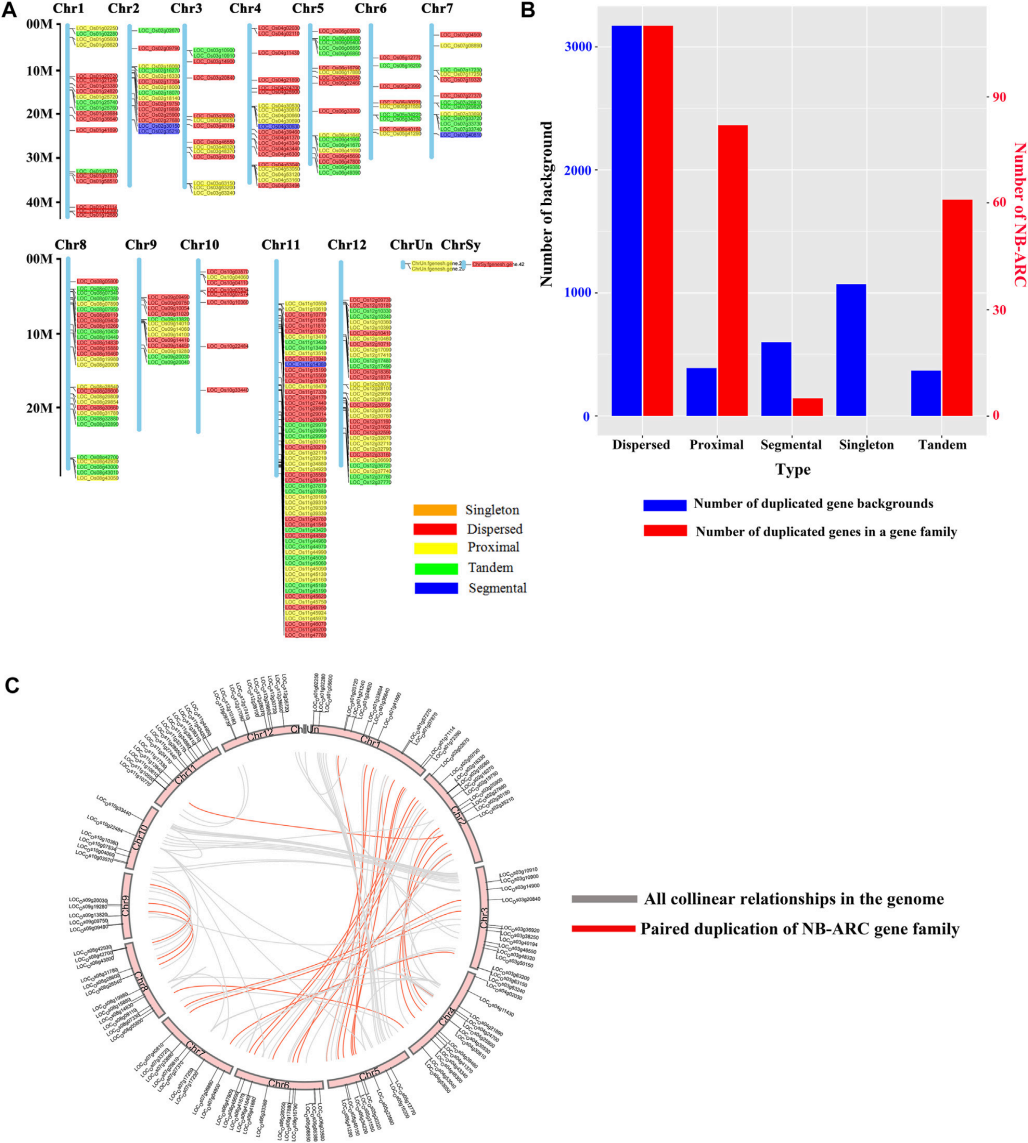
Chromosomal distribution of NB-ARC gene duplication events. **(A)** Distribution of NB-ARC genes with indicated duplication types. **(B)** Numbers of duplicated genes for different duplication types. **(C)** Collinearity of replicative genes in the protein family.

A total of 258 rice NB-ARC genes were identified in the *Nipponbare* genome (Supplementary Table S2). The lengths of the CDS for these genes range from 624 (*LOC_Os02g27680*) to 7,773 (*LOC_Os06g41690*) bp. Analysis revealed that 49.22% of these NB-ARC genes contain 2–6 introns and 86.04% NB-ARC genes contain 1–4 exons (Supplementary Table S2). Most of the NB-ARC genes have a long CDS and show a highly conserved structure (Figure 1). To explore the expansion of NB-ARC family members in rice, these sequences were used to generate an unrooted phylogenetic tree. The NB-ARC family members were divided into three major groups. Group I contained 29 genes, Group II contained 226 genes, and group III contained only three genes. The NB-ARC genes in group II were further classified into nine subgroups (groups IIa, IIb, IIc, IId, IIe, IIf, IIg, IIh, and IIi). Of these, the two groups with the most members were IIf with 35 genes and IIi with 37 genes. Group IIc contained the fewest number of genes, 13. A total of 15 motifs were identified by MEME, and two of these, M2 and M6, were present in 98.83 and 97.28%, respectively, of the NB-ARC genes. Genes on a single branch contained similar numbers of introns and similar distribution and number of motifs. In a given subtribe of genes, the positions of motifs were highly conserved. In group IIf, the genes contained 1-4 introns and 2–12 exons. Most genes in this group share 10 similar motifs (motifs 1, 2, 3, 4, 6, 8, 9, 10, 11, and 12) (Figure 1). Group IIf was representative in motifs and numbers of NB-ARC genes. Overall, NB-ARC genes were unevenly distributed across all 12 chromosomes, with 29 (11.24%), 61 (23.64%), and 34 (13.18%) genes located on Chr 8, 11, and 12, respectively. (Figure 2A). Gene analysis revealed duplication events including segmental, tandem, proximal, and dispersed, but not singleton duplications. Dispersed (110) and proximal replication 82) most frequently occurred in NB-ARC genes across all chromosomes (Figure 2B). Segmental replication was detected only on Chr2, Chr4, Chr7, and Chr11 (Figure 2B). Pairwise replication of genes was also detected in NB-ARC genes (Figure 2C). The genes in Group IIf, mapped to eight chromosomes, with dispersed, tandem, and proximal replication. In general, genes in subgroup IIf of group II showed typical structure with main motif and contain more genes, and analysis suggested that replication events were the main driving force of NB-ARC evolution.

### Conservation of NB-ARC Homologous Genome Segments in Monocotyledons With Weak Positive Selective Pressure During Evolution

**FIGURE 3:**
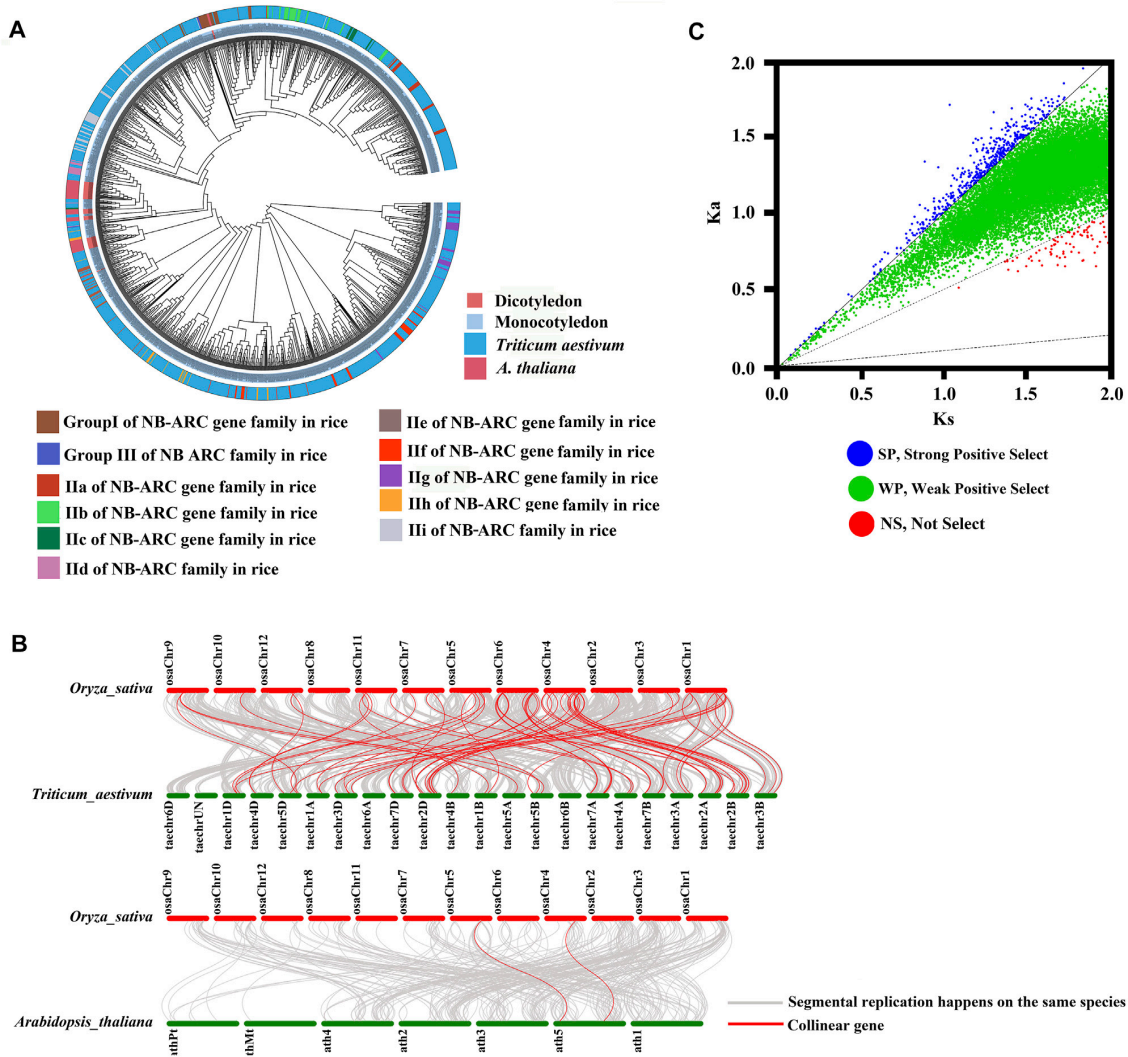
Phylogenetic, Ka/Ks, and synteny analysis of NB-ARC genes from rice, *Arabidopsis,* and *Triticum aestivum*. **(A)** Group I, Group II, Group III, IIa, IIb, IIc, IId, IIe, IIf, IIg, IIh, and IIi were identified in the rice genome; 1,052 and 50 NB-ARC genes were identified in *Triticum aestivum* and *Arabidopsis thaliana*. Genes from rice, *Arabidopsis*, *Triticum aestivum* are indicated with the prefixes Os, At, and Tran, respectively. **(B)** Synteny analysis of *Oryza sativa*, *Arabidopsis*, and *Triticum aestivum.* Gray lines in the background indicate collinear blocks of plant genomes. Different color bars represent the chromosomes of different species. The chromosome number is labeled at the top or bottom of each chromosome. **(C)** Ka/Ks analysis of NB-ARC genes from rice, *Arabidopsis* and *Triticum aestivum*.

To study the relationships between the NB-ARC genes of rice and other model plant species, including gramineous plants, we performed cluster analysis (Figure 3A). The NB-ARC genes of a dicotyledon plant, *Arabidopsis*, and two monocotyledonous plants, rice and wheat, were compared. Pfam was used to compare 1,052 and 50 NB-ARC genes in wheat and *Arabidopsis*, respectively. Next, based on the highly conserved NB-ARC domains of rice, wheat*,* and *Arabidopsis*, a phylogenetic tree was built. The 50 genes of *Arabidopsis* belonged to a single subgroup, and every subgroup contained rice and wheat genes (Figure 3A). Within each subgroup, genes from the homologous chromosome group generally clustered into a clade (Figure 3A); for example, *LocOs12g36690*, *LocOs12g36720*, and *LocOs12g33160* were clustered in a subgroup. To further study the relationships between the NB-ARC genes of rice and other plants, we next performed whole genome synteny analysis (Figure 3B). A total of 65 homologous genome segments were distributed on 12 chromosomes in wheat (Supplementary Table S3). The highest number of homologous genome segments, 19, mapped to the syntenic locus in chromosome 4 and wheat chromosomes 2A, 2B, 2D. Two homologous genome segments mapped to chromosome 5 in *Arabidopsis* (Figure 3B) For further evolutionary analysis, the Ka, Ks, and Ka/Ks values of homologous gene pairs were calculated based on the comparative synteny map (Figure 3C). Ka/Ks ratios of 2,571 gene pairs were evaluated in rice and wheat in protein coding genes. Fifty-two gene pairs had Ka/Ks > 1, 2,412 gene pairs had 0.5 < Ka/Ks < 1, and no gene pairs had <0.1. Ka/Ks ratios were similarly evaluated for 70 gene pairs in rice and *Arabidopsis thaliana* to examine in protein coding genes. Five gene pairs had Ka/Ks > 1, 59 gene pairs had 0.5 < Ka/Ks < 1, no gene pairs had <0.1, and the remaining had no data so could not be calculated. In rice, 15 gene pairs had Ka/Ks > 1, 431 gene pairs had 0.5 < Ka/Ks < 1, no gene pairs had <0.1, and five gene pairs had 0.1 < Ka/Ks < 0.5 (Supplementary Table S4). Because the majority of homologous NB-ARC gene pairs had 0.5 < Ka/Ks < 1, this suggests that the NB-ARC gene family in these three plant species experienced weak positive selective pressure during evolution. Evolution of the few NB-ARC genes in rice was governed by strong constraints that may have contributed to their structural and functional stability (Figure 2C and Supplementary Table S5). Interestingly, some homologous genome segments mapped between rice and wheat were not observed between rice and *Arabidopsis*, which may indicate that these homologous pairs formed after the divergence of dicotyledonous and monocotyledonous plants. Overall, the analysis shows that the NB-ARC gene family is highly conserved in monocotyledons and homologous genome segments suggest that segmental duplications may be the main cause of the extension of this gene family. Multiple copies of genes have arisen during the evolution of dicotyledons and monocotyledons.

### Most NB-ARC Genes Regulate Panicle Development

**FIGURE 4:**
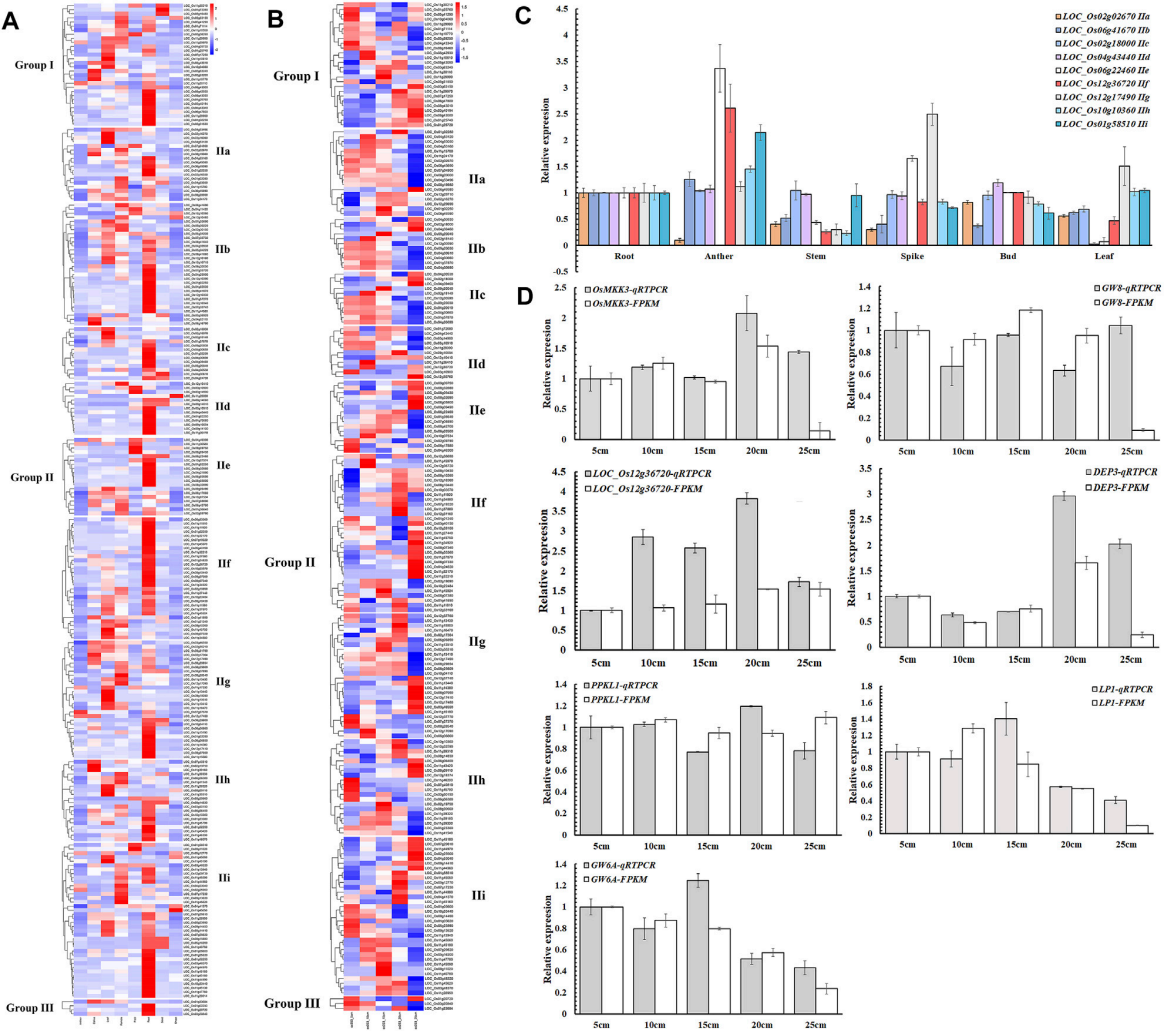
Expression profiles of NB-ARC genes. **(A)** Expression patterns of rice NB-ARC genes. The expression data are RNA-Seq data from the Rice Expression Database. The white box indicates no expression (zero fragments per kb of exon per million mapped reads (FPKM)) in this tissue. The transcript abundances in different tissues in the heat map were estimated by Log2 (FPKM) values. **(B)** Expression patterns of rice NB-ARC genes in Ce253 at 5, 10, 15, 20, and 25 cm panicle lengths. **(C)** Expression of rice NB-ARC genes in root, anther, shoot, bud, leaf, and panicle of Ce253. **(D)** Expression of key genes that control panicle development in 5, 10, 15, 20, and 25 cm panicle lengths in Ce253. Data are given as mean ± s. e.m. (n = 3). *UBQ* (ubiquitin) was used as a control. Error bar indicates 95% confidence interval.

To study the spatial and temporal expression patterns of NB-ARC genes, transcriptomic profiling was profiled across eight different tissues (anther, callus, leaf, panicle, pistil, root, seed, and shoot) to analyze the role of these genes in rice organ development according to the Rice Expression Database (Supplementary Table S6 and Figure 4A). In our findings, 23 genes exhibited no expression in any tissues, five genes showed expression in eight tissues, and the remaining 88 genes showed FPKM values higher than 1 in more than three tissues. *LOC_Os02g25900* and *LOC_Os12g36720* exhibited FPKM values higher than 1 in anther, callus, leaf, panicle, pistil, root, seed, and shoot. Expression of these genes varied for different tissues, with expression of 141 genes in root, 94 genes in panicle, and only nine genes in anther.

For the IIf subgroup, one gene was most expressed in callus and 14 genes were most expressed in panicle. Six genes of IIf were most expressed in pistil and 23 genes of IIf were most expressed in root. IIf, a typical subgroup in the NB-ARC gene family, includes genes such as *LOC_Os12g36720* that was expressed in eight different tissues (Figure 4A). To confirm the expression patterns, expression levels of 11 genes of different subgroups were detected by qRT-PCR. The results showed that six of these genes exhibited similar expression patterns to the transcriptomic data (Figure 4B). Most of these genes showed high expression in root. *LOC_Os12g36720* in IIf was most highly expressed in anther and panicle.

Few studies have explored the roles of NB-ARC genes in normal plant growth and development. To investigate a possible function of these genes to regulate plant panicle development, the expression of selected NB-ARC genes was analyzed at the panicle development stage of cultivated rice (Figure 4B, c). In rice Ce253, 40, 34,32, 46, and 64 genes exhibited FPKM values higher than 1 at 5, 10, 15, 20, and 25 cm panicle length stages, respectively, and 215, 221, 223, 209, and 191 genes had FPKM values lower than 1 in 5, 10, 15, 20, and 25 cm panicle length stages, respectively. Most genes of the first subgroup of IIf had FPKM values lower than 1 at the early stage of young panicle differentiation (5, 10 cm), 12 and 11 genes exhibited FPKM values higher than 1 at 20 and 25 cm panicle length stages, respectively, and two, four, and four genes at 5, 10, and 15 cm panicle length stages, respectively (Figure 4B). The observed expressional activation in panicle development for *LOC_Os12g36690*, *LOC_Os11g45970*, and *LOC_Os12g36720* suggests these genes play a crucial role in panicle development of rice.

### *GNP12* Regulates Rice Yield With Effects on Panicle Length, Panicle Grain Number, and Grain Length

**FIGURE 5:**
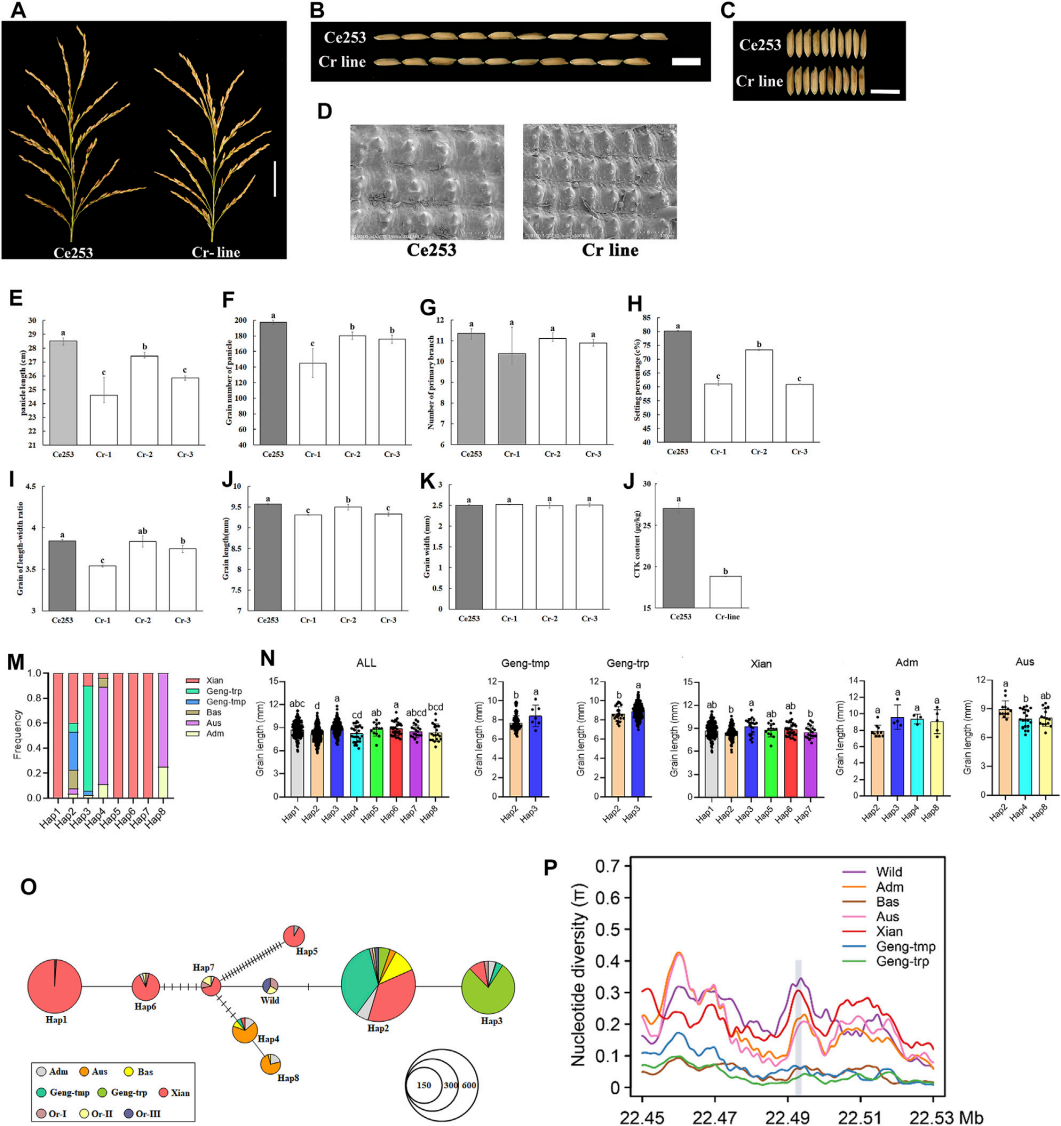
The effect of *GNP12* on cell proliferation length of panicle, grain number of panicle and grain size. **(A)** Length of panicle of Ce253 and Cr-lines. Scale bars, 5 cm. **(B)** Length of grains from Ce253 and Cr-line. **(C)** Width of grains from Ce253 and Cr-line. Scale bars, 1 cm. **(D)** Scanning electron microscopic images of chalkiness in transgenic plants and Ce253. Bar, 50 μm. **e-l** Panicle length (cm) **(E)**, grain number of panicle **(F)**, primary branch number **(G)**, setting percentage (%) **(H)**, grain of length-width ratio **(I)**, grain length (mm) **(J)**, grain width (mm) **(K)**, CTK content in Ce253 and Cr-line **(l)**. Data are given as mean ± s. e.m. (n = 12). Error bar indicates 95% confidence interval. Student’s *t* tests were used to generate *p* values. Haplotype and evolutionary analysis of *GNP12* in rice **(M)** Major haplotypes of *GNP12* using 32 single-nucleotide polymorphisms (SNPs) in the coding sequence region in 827 accessions **(N)** Phenotypic evaluation of haplotype grain length in different subgroups **(O)** Haplotype network analysis of *GNP12* **(P)** Nucleotide diversity of *GNP12.*

To test the ability of NB-ARC genes to regulate plant panicle development, *LOC_Os12g36720* was selected as a representative IIf gene. Expression of this gene was detected in anther, callus, leaf, panicle, pistil, root, seed, and shoot, and exhibited a remarkably high level of expression in Ce253 during panicle development. Interestingly, *LOC_Os12g36720* encodes *GNP12*, a *Resistance Gene Homologs* (*RGH1A*) gene (Supplementary Table S7). Therefore, *LOC_Os12g36720, GNP12*, was selected for further characterization (Supplementary Table S6). To further characterize the function of this gene, we used a CRISPR-Cas9 system for targeted gene mutation of *GNP12* in Ce253 (Cr line) (Figure 5A). The resulting deletions lead to frameshifting mutations that result in incomplete peptides of *GNP12* that exhibit a loss of NB-ARC domain function. Compared to Ce253, the three Cr lines Cr-1, Cr-2, and Cr-3, were reduced 13.68, 3.71, and 9.39%, respectively, in panicle length (Figure 5B); 23.80, 8.50, and 24%, respectively, in panicle grain number; 2.76, 0.7, and 2.51%, respectively, in grain length; and 26.58, 8.79, and 10.98% in setting percentage, respectively. The three Cr lines showed significant reductions in panicle length, grain number of panicle, grain length, and setting percentage (Figure 5A, b, e, f, h, j). To investigate the tissue structures affected by *GNP12,* microstructures of grain chaff were observed by scanning electron microscopy (Figure 5D). The chaff of Cr was shorter than that of Ce253 (Figure 5D), but the Cr lines did not differ in length-width grain ratio or grain width (Figure 5C, i, k).

Cytokinin (CTK) is a primary determinant of plant architecture (Tameling et al., 2002). To investigate whether *GNP12* influences CTK levels, we compared the CTK content in wild-type and Cr lines. The CTK content in Cr lines was reduced to 30.28% of that in the wild-type (Figure 5l). The results suggest that *GNP12* may affect panicle development and grain production in rice through changes in CTK content.

To investigate natural variation in *GNP12*, we analyzed the polymorphism of *GNP12* in the 3K rice genomes (3K-RG) dataset (Wang et al., 2018). We identified eight major haplotypes of *GNP12* with 32 single-nucleotide polymorphisms (SNPs) in the coding sequence region in 827 accessions of the 3K-RG dataset (Figure 5m and Table 1). Except for Hap2, the frequencies of the major haplotypes differed significantly among different subgroups. Hap1, Hap5, Hap6, and Hap7 were specifically found in *indica* rice cultivars, with Hap1 being the most prevalent. Hap2 was mainly identified in *indica*, temperate *japonica*, tropical *japonica,* and Bas rice cultivars. Hap3 was mainly present in tropical *japonica* and *indica* rice cultivars. Hap4 and Hap8 were mainly found in *Aus* rice cultivars (Figure 5n). Phenotypic evaluation showed that Hap1, Hap3, Hap5, and Hap6 in the *indica* subgroup and Hap3 in the *japonica* subgroup showed longer grain length than other haplotypes. Haplotype network analysis showed that favorable haplotypes of *GNP12* may have different origins. Favorable *indica* haplotypes Hap3 and Hap6 may have come directly from *O. rufipogon* I and favorable *indica* haplotypes Hap1 and Hap5 may have come from novel favorable mutations during *indica* domestication. Favorable haplotype Hap3 in *japonica* may have arisen during the differentiation of temperate and tropical *japonica* (Figure 5o).

**TABLE 1:** Haplotype analysis of GNP12.

	Hap1	Hap2	Hap3	Hap4	Hap5	Hap6	Hap7	Hap8
22,488,973	T	T	T	T	G	T	T	T
22,488,980	T	T	T	T	C	T	T	T
22,489,046	A	A	A	A	C	A	A	A
22,489,052	A	A	A	A	T	A	A	A
22,489,099	C	C	A	C	C	C	C	C
22,489,112	T	T	T	T	C	T	T	T
22,489,124	G	G	G	G	C	G	G	G
22,489,273	T	T	T	T	A	T	T	T
22,489,347	G	T	T	T	G	G	T	T
22,489,410	A	T	T	T	T	A	T	T
22,489,411	A	C	C	C	C	A	C	C
22,489,412	A	T	T	T	T	A	T	T
22,489,447	G	G	G	G	A	G	G	G
22,489,482	A	A	A	A	T	A	A	A
22,489,489	C	C	C	C	T	C	C	C
22,489,499	G	G	G	G	T	G	G	G
22,489,559	T	C	C	C	C	T	C	C
22,489,568	T	C	C	C	T	T	C	C
22,489,600	C	A	A	A	C	C	C	A
22,489,759	G	G	G	G	T	G	G	G
22,489,794	T	T	T	T	G	T	T	T
22,489,825	C	C	C	C	T	C	C	C
22,489,831	T	T	T	T	C	T	T	T
22,489,832	G	G	G	G	A	G	G	G
22,489,838	C	C	C	C	A	C	C	C
22,489,841	T	T	T	T	C	T	T	T
22,490,008	T	T	T	C	T	T	T	C
22,490,063	C	C	C	C	C	C	C	T
22,490,105	A	A	A	C	C	A	A	C
22,490,129	T	T	T	G	G	T	T	G
22,490,179	C	C	C	T	C	C	C	T
22,491,343	T	T	T	C	C	C	C	T
All	178	320	220	27	13	29	20	20
Adm	1	11	5	3	0	0	0	5
Aus	0	13	0	21	0	0	0	15
Bas	0	49	0	2	0	0	0	0
Geng-tmp	0	97	7	0	0	0	0	0
Geng-trp	0	23	186	0	0	0	0	0
Xian	177	127	22	1	13	29	20	0

To determine whether selection acted on *GNP12*, we analyzed the nucleotide diversity of *GNP12* among different rice subgroups. On average, the nucleotide diversities of *GNP12* in tropical *japonica* and *indica* subgroups were respectively much lower and higher than those in other haplotypes. To assess whether the DNA sequence evolved randomly or under a non-random process, Tajima’s D values were calculated for *GNP12* in tropical *japonica* and *indica* and were negative and positive, respectively. Both values deviated significantly from zero, implying potential positive selection and balancing selection acting on *GNP12* in tropical *japonica* and *indica,* respectively (Figure 5p). To further test whether the observed reduction in nucleotide diversity in tropical *japonica* was due to positive selection or a bottleneck effect, we calculated the nucleotide diversity of 40-kb flanking regions of *GNP12.* We found that the average nucleotide diversity of the *GNP12* flanking regions in tropical *japonica* was significantly lower than that in other regions, suggesting that the decrease of nucleotide diversity of *GNP12* in tropical *japonica* may be largely caused by positive selection (Table 2).

**TABLE 2:** Pi and Tajima’s D of GNP12.

Region	Parameter	Adm (94)	Aus (109)	Bas (75)	Xian (1,487)	Geng-Tmp (270)	Geng-Trp (300)	Geng (570)	Cultivated (2,335)	Wild Rice (47)
Upstream 40 kb	S	417	267	242	506	251	198	257	520	416
	π	0.13228	0.15248	0.03796	0.19926	0.03453	0.02692	0.03253	0.16843	0.168
	θ	0.15647	0.09736	0.09503	0.12323	0.09219	0.06052	0.0825	0.11978	0.15491
	Tajima’s D	−0.52663	1.89298	−2.07817*	1.7679	−1.94536*	−1.70608	−1.7997	1.14314	0.30965
*RGH1A*	S	17	15	11	16	18	14	18	18	29
	π	0.28385	0.1991	0.06583	0.284	0.10599	0.02777	0.06628	0.2748	0.32441
	θ	0.18464	0.15831	0.12502	0.12688	0.16198	0.13115	0.14447	0.12001	0.18239
	Tajima’s D	1.53066	0.70509	−1.28911	2.68133*	−0.88196	−1.89673*	−1.27709	2.8384*	1.87248
Downstream 40 kb	S	420	314	360	479	402	347	405	489	460
	π	0.20758	0.18333	0.05493	0.20429	0.08986	0.04999	0.07115	0.23071	0.21806
	θ	0.15405	0.11605	0.12831	0.12253	0.11344	0.09627	0.10193	0.11855	0.17358
	Tajima’s D	1.18392	1.94233	−1.98862*	1.9106	−0.6511	−1.49276	−0.90395	2.66024*	0.94009

### NB-ARC Proteins Contain Conserved P-Loop Domains

**FIGURE 6:**
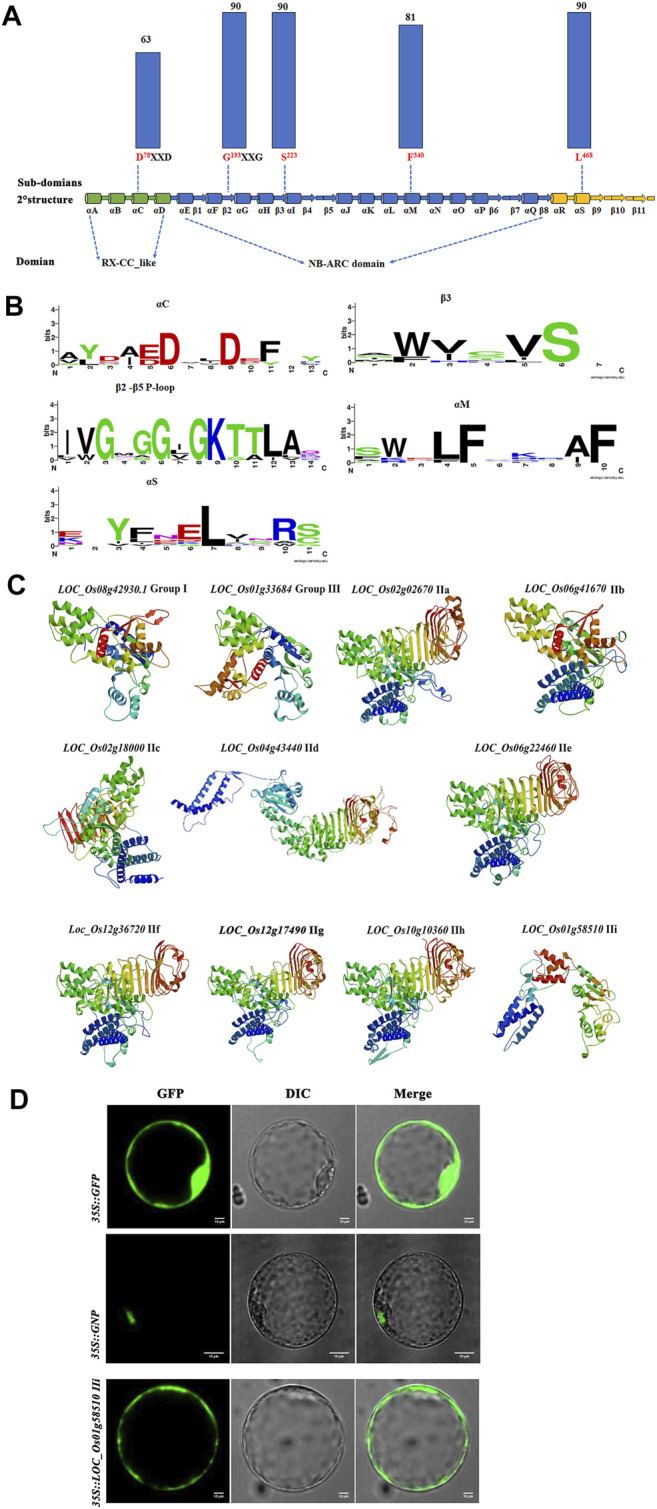
Predicted structures and subcellular localization of putative NB-ARC protein domains based on 11 proteins from each 11 subgroups. **(A)** Conservation of key motifs, residues, and secondary structure between putative NB-ARC domains. Dashed lines indicate positions within secondary structure elements. The histograms above the motifs represent the degree of conservation (% of identical to conserved residuals) for putative NB-ARC domains (blue columns) among 11 proteins from 11 subgroups. **(B)** Sequence logos representing the conservation of key motifs and neighboring sequences. The size of the letters corresponds to information content. **(C)** 3D model of 11 proteins from 11 subgroups in NB-ARC family. **(D)** Subcellular localization of three proteins from groups I, IIf, and IIi.

To further investigate the subdomains and functions of NB-ARC genes, 11 predicted proteins from group I, group II, and group III were selected for further analysis. Strong conservation was identified for several subdomains: αC, αM, αS, β3, β2-β5, and P-loop (Figure 6A, b). The most conserved residues in the putative NB-ARC domains were found in the P-loop (G193 and S223) and αS (L468) subdomains (Figure 6A). Analysis of the gene structure and conserved motifs revealed high conservation of rice NB ARC genes with no obvious variation in the amino acid residues of the P-loop region among homologous genes (Figure 6C). We observed few variants of the 11 amino acids in the P-loop core, with substantial variation in genic frequency of these variants in rice. These results indicate the significant conservation of the P-loop in most rice NB-ARC proteins. The predicted 3D structures of the identified rice NB-ARC proteins were conserved, which was consistent with the phylogenetic, gene structure, and conserved domain analysis. *LOC_Os02g02670*, *LOC_Os06g41670*, *LOC_Os06g22460*, *LOC_Os012g36720*, *LOC_Os12g17490.1*, and *LOC_Os10g10360* were predicted to share similar structures (Figure 6C). Predicted models were constructed to heuristically maximize alignment coverage, identity percentage, and confidence score for the tested sequences. For the 11 predicted NB-ARC proteins, α-helix was most highly predicted as secondary structure (37.41–63.56%), followed by random coil (20.03–35.64%), extended strands (7.3–20.84%) and β-strands (0–5.29%) (Figure 6C and Supplementary Table S8). The 3D modeling results revealed similarity of predicted tertiary structures, implying that NB-ARC proteins may have evolved from a shared ancestor and/or under purification selection force for stabilization during long-term acclimation after initial divergence.

To further characterize the function of the NB-ARC genes, subcellular localization studies were performed. We randomly selected two NB-ARC genes, *LOC_Os12g36720* (IIf), *LOC_Os101g458510* (IIi), for subcellular localization in rice protoplast cells. *LOC_Os12g36720*-GFP and *LOC_Os101g458510*-GFP expression vectors were constructed and transformed into rice protoplasts for transient expression. Control GFP expression was observed throughout the membrane, cytoplasm and nucleus, and the *GNP12* and *LOC_Os101g458510* proteins were mainly expressed in the nucleus and membrane, respectively. This result indicated that these proteins are widely present in various organelles.

## Discussion

### Conserved Domains and Polymorphisms of NB-ARC Genes Maintained a Dynamic Balance During Evolution

NB-ARC proteins play central roles in recognizing pathogens, initiating defense cascades, and maintaining cell development. The NB-ARC conserved domain maintains stable similar structures in different species, likely due to convergent evolution, suggesting these proteins may perform similar recognition and activation mechanisms (Slootweg et al., 2009). In the Fabaceae family, eight conserved motifs of NB-ARC domains have been identified (Pal et al., 2007). Plant NOD-like receptor (NLR) proteins contain NB-ARC domains with structural similarities to their mammalian homologues (Steele et al., 2019). However, there is high polymorphism in some NB-ARC genes in some species. The combination of conserved motifs and the evolution of rich polymorphisms may allow response to environmental signal stimulation to achieve coevolution by replication events. In *Arabidopsis*, analysis of the NLR family revealed that positive selection and recombination occurred frequently in the leucine-rich repeat (LRR) domain but there was negative selection in the nucleotide-binding (NB-ARC) domain (Mondragón-Palomino et al., 2017). In peanut, *AhRAF4* of NB-ARC proteins evolved by recombination with duplications and point mutations from *Arachis duranensis* (Deng et al., 2018). Among *O. sativa* (Slootweg et al., 2009), *O. glaberrima* (Slootweg et al., 2009), and *O. brachyantha* (FF), a high number of paralogs suggests that the NB-ARC family experienced highly dynamic evolution, with a large number of tandem arrays and duplicated genes observed in the *O. sativa* subspecies (Jacquemin et al., 2014). NB-ARC genes diversified through duplication to encode receptors adapted to external signals (Andersen et al., 2020). IPm21 encodes a coiled-coil, nucleotide-binding site, leucine-rich repeat (CC-NBS-LRR) protein, and evolutionary analysis of 38 non-redundant Pm21 alleles indicated that the nucleotide diversity of the LRR domain was significantly higher than those of the CC and NB-ARC domains (He et al., 2020). NB-ARC genes can form genomic clusters adjacent to LRR-RLK-XIIs by mechanisms other than genomic clustering (Ngou et al., 2022). Overall, the evolution of NB-ARC genes allows these genes to maintain key function through conservation of the NB-ARC domain while the C-terminal regions of these proteins exhibit polymorphisms to allow these genes to co-evolve with the environment through replication events.

### *GNP12* May Regulate Rice Yield by Influencing Hormone Activity

Panicle grain number and length of panicle are important agronomic characteristics, but the genetic determinants of these traits remain unclear. Many genes regulate grain number per panicle, length of panicle, and grain length by influencing hormone activity. *ERECTA1* (*OsER1*) is a cytokinin oxidase/dehydrogenase that negatively regulates grain number per panicle (Ashikari et al., 2005). *ERECTA1* acts upstream of the *OsMKKK10*-*OsMKK4*-*OsMPK6* cascade, and the *OsER1*-*OsMKKK10*-*OsMKK4*-*OsMPK6* pathway is required to maintain cytokinin homeostasis (Guo et al., 2020). *OsASP1* encodes a TOPLESS-related transcriptional co-repressor, is closely associated with auxin action, and regulates spikelet development (Yoshida et al., 2012). *TGW6* encodes a protein with indole-3-acetic acid (IAA)-glucose hydrolase activity that can control IAA supply at the transition from the syncytial to the cellular phase to limit cell number and grain length (Ishimaru et al., 2013). *GW6* encodes a GA-regulated GAST family protein and positively regulates grain width and weight (Shi et al., 2020).

NB-ARC genes regulate plant development by catalyzing ADP through ARC structure. NB-ARC proteins carry a central nucleotide-binding-ARC domain that binds ADP/ATP rather than GDP/GTP (Chattopadhyaya and Pal, 2008). The NB-ARC domain may be a molecular switch between ADP (repressed) and ATP (active) binding forms (Steele et al., 2019). A tomato (*Lycopersicon esculentum*) R protein I-2 with NB-ARC domain is impaired in ATP hydrolysis, but not in ATP binding, suggesting a molecular switch whose state (on/off) depends on nucleotide binding (ATP/ADP) (Tameling et al., 2006). *NRTP1* encodes a CC-NB-LRR type protein, and semi-dominant mutant nrtp1-D contains an amino acid substitution in the NB-ARC domain that causes constitutive auto-activation of the NRTP1 protein for a short-root phenotype in rice (Yu et al., 2018). ATP/ADP opentenyltransferases are likely responsible for most isopentenyladenine- and tZ-type cytokinin synthesis (Miyawaki et al., 2006) and catalyze prenylation of adenosine diphosphate (ADP) or triphosphate (ATP) biosynthesis (Kieber and Schaller, 2014). The hydrolysis of adenosine triphosphate (ATP) is directly coupled to the primary active transport of CTK (Nedvěd et al., 2021). In peach, ATP/ADP PpIPT genes are key genes for cytokinin biosynthesis in nodal stems (Li et al., 2018).

The NB-ARC domain of *GNP12* protein may act in panicle development by influencing hormone activity to control grain number of panicle, length of panicle, and grain length. Future work is required to test this hypothesis and investigate the regulation of these important genes to better develop molecular tools for improved genetic breeding.

## Conclusion

In summary, we successfully analyzed the NB-ARC family of genes with conserved P-loop domains and identified conservation of NB-ARC homologous genome segments in monocotyledons. Most NB-ARC genes regulate panicle development in Ce253. We identified *GNP12* as an important regulator of panicle length, panicle grain number, and grain length.

## Data Availability

The original contributions presented in the study are publicly available. This data can be found here: https://www.ncbi.nlm.nih.gov/ PRJNA815477.

## Abbreviations

Chr: chromosome; CTK: Cytokinin; GNP: Grain Number per Panicle; IAA: Indole-3-acetic acid; MEME: Multiple EM for motif elicitation; NB-ARC: nucleotide-binding adaptor shared by APAF-1, R proteins and CED-4; ORF: Open reading frame; qRT-PCR: quantitative Reverse-Transcription Polymerase Chain Reaction; SNPs: Single-nucleotide polymorphisms.
